# NLRC4 and TLR5 Each Contribute to Host Defense in Respiratory Melioidosis

**DOI:** 10.1371/journal.pntd.0003178

**Published:** 2014-09-18

**Authors:** T. Eoin West, Nicolle D. Myers, Narisara Chantratita, Wirongrong Chierakul, Direk Limmathurotsakul, Vanaporn Wuthiekanun, Edward A. Miao, Adeline M. Hajjar, Sharon J. Peacock, H. Denny Liggitt, Shawn J. Skerrett

**Affiliations:** 1 Division of Pulmonary & Critical Care Medicine, Department of Medicine, University of Washington School of Medicine, Seattle, Washington, United States of America; 2 International Respiratory and Severe Illness Center, University of Washington, Seattle, Washington, United States of America; 3 Department of Microbiology and Immunology, Faculty of Tropical Medicine, Mahidol University, Bangkok, Thailand; 4 Mahidol-Oxford Tropical Medicine Research Unit, Faculty of Tropical Medicine, Mahidol University, Bangkok, Thailand; 5 Department of Clinical Tropical Medicine, Faculty of Tropical Medicine, Mahidol University, Bangkok, Thailand; 6 Department of Tropical Hygiene, Faculty of Tropical Medicine, Mahidol University, Bangkok, Thailand; 7 Department of Microbiology and Immunology, Lineberger Comprehensive Cancer Center, and Center for Gastrointestinal Biology and Disease, University of North Carolina at Chapel Hill, Chapel Hill, North Carolina, United States of America; 8 Department of Comparative Medicine, University of Washington School of Medicine, Seattle, Washington, United States of America; 9 Department of Medicine, University of Cambridge, Addenbrooke's Hospital, Cambridge, United Kingdom; University of Tennessee, United States of America

## Abstract

*Burkholderia pseudomallei* causes the tropical infection melioidosis. Pneumonia is a common manifestation of melioidosis and is associated with high mortality. Understanding the key elements of host defense is essential to developing new therapeutics for melioidosis. As a flagellated bacterium encoding type III secretion systems, *B. pseudomallei* may trigger numerous host pathogen recognition receptors. TLR5 is a flagellin sensor located on the plasma membrane. NLRC4, along with NAIP proteins, assembles a canonical caspase-1-dependent inflammasome in the cytoplasm that responds to flagellin (in mice) and type III secretion system components (in mice and humans). In a murine model of respiratory melioidosis, *Tlr5* and *Nlrc4* each contributed to survival. Mice deficient in both *Tlr5* and *Nlrc4* were not more susceptible than single knockout animals. Deficiency of *Casp1/Casp11* resulted in impaired bacterial control in the lung and spleen; in the lung much of this effect was attributable to *Nlrc4*, despite relative preservation of pulmonary IL-1β production in *Nlrc4^−/−^* mice. Histologically, deficiency of *Casp1/Casp11* imparted more severe pulmonary inflammation than deficiency of *Nlrc4*. The human *NLRC4* region polymorphism rs6757121 was associated with survival in melioidosis patients with pulmonary involvement. Co-inheritance of rs6757121 and a functional *TLR5* polymorphism had an additive effect on survival. Our results show that NLRC4 and TLR5, key components of two flagellin sensing pathways, each contribute to host defense in respiratory melioidosis.

## Author Summary

Melioidosis is an infection caused by *Burkholderia pseudomallei*, a bacterium that is found in tropical soil and water. Melioidosis can present in a variety of ways, but lung involvement is common and usually severe. The host response to infection governs outcome. In this study, we examined the role of two host sensors of bacterial components–TLR5 and NLRC4–to determine their necessity in respiratory melioidosis. Although both proteins are involved in detection of bacterial flagellin, in mice we defined specific and individual roles for TLR5 and NLRC4 in protecting against death from melioidosis. In humans with melioidosis involving the lung, genetic variation in these receptors also had independent associations with survival. These results underscore the importance of these elements of host defense in respiratory melioidosis and support further studies of the underlying mechanisms.

## Introduction

*Burkholderia pseudomallei* is a tropical soil saprophyte and Tier 1 select agent that causes the infection melioidosis [1]. The bacterium may be inoculated through the skin, inhaled, or ingested. Although infection can manifest in myriad ways, pneumonia is identified in 50% of cases. Mortality from melioidosis ranges from 14–40% despite appropriate antibiotic treatment, and the risk of death is higher with pulmonary involvement [2], [3]. This indicates an urgent need for a better understanding of host-pathogen interactions in melioidosis and adjunctive immuno-modulatory therapies.

Innate immune mechanisms of recognition of invading bacteria include membrane-bound Toll-like receptors (TLRs) and cytosolic NOD-like receptors (NLRs) [4], [5]. These pathogen recognition receptors bind conserved pathogen associated molecular patterns and drive the host response. For example, as a Gram-negative, flagellated bacterium, *B. pseudomallei* is predicted to activate sensors of LPS (such as TLR4) and flagellin (such as TLR5). We have found that *B. pseudomallei* LPS is a TLR4 ligand that drives much of the innate immune response to *B. pseudomallei*, and that human genetic variation in *TLR4* is associated with susceptibility to melioidosis [6]–[8]. We have also shown that *B. pseudomallei* activates TLR5, and that polymorphisms in *TLR5* are associated with survival from melioidosis [9], [10], however TLR5-deficient mice have not been infected with *B. pseudomallei* to demonstrate the role of TLR5 in an experimental setting. These findings point to an important role for flagellin in activation of immune responses in melioidosis.

Whereas TLR5 detects flagellin at the cell surface, cytosolic flagellin is detected through NLRC4, an inflammasome that activates caspase-1 [11]. NLRC4 is one of a number of NLRs that can assemble a canonical caspase-1-dependent inflammasome that in turn cleaves pro-IL-1β and pro-IL-18 to their active forms and induces pyroptosis [5], [12]. More recent work identified murine NAIP5 and NAIP6 as direct flagellin sensors that signal through NLRC4 [13], [14]. NLRC4 also contributes to the sensing of bacterial components other than flagellin: murine NLRC4-NAIP1 and NLRC4-NAIP2 inflammasomes recognize bacterial type three secretion system (T3SS) needle and rod proteins, respectively [13]–[16]. In contrast to mice, humans have only a single NAIP, and in human U937 monocytes the NLRC4-NAIP inflammasome recognizes a T3SS needle protein but not flagellin [14]. The functional interpretation of the three NLRC4 agonists is similar – flagellin, rod, and needle protein are all believed to be accidentally injected into the cytosol by bacterial T3SS. This is in contrast to TLR5 detecting extracellular flagellin, the presence of which will not be strictly linked to a particular virulence trait. In addition to the canonical caspase-1-dependent inflammasome, a noncanonical inflammasome involving another inflammatory caspase, caspase-11, has recently been described in mice [17]. Caspase-11 protects mice from *B. pseudomallei* infection [18].

In this study, our primary objective was to determine the relative importance of NLRC4 in murine respiratory melioidosis in comparison to TLR5, and with respect to canonical and noncanonical inflammasomes. Our secondary objective was to test whether genetic variation in NLRC4 was associated with outcome in human respiratory melioidosis.

## Methods

### Ethics statement

All animal experiments were approved by the University of Washington Institutional Animal Care and Use Committee (protocol number 2982-03). The University of Washington complies with all applicable provisions of the federal Animal Welfare Act and with the Public Health Service (PHS) Policy on Humane Care and Use of Laboratory Animals. The University of Washington Human Subjects Division Institutional Review Board; the Ethical Review Committee for Research in Human Subjects, Ministry of Public Health, Thailand; and the Ethics Committee of the Faculty of Tropical Medicine, Mahidol University, Bangkok, Thailand approved the human genetic studies on subjects who had provided or whose next of kin had provided written informed consent for enrollment into clinical studies of melioidosis at the time of recruitment.

### Bacteria

*B. pseudomallei* 1026b was grown in LB broth shaking in air at 37°C, washed twice, resuspended in PBS containing 20% glycerol, and frozen at −80°C. Immediately before each aerosol infection experiment, the freezer stock was thawed and diluted in PBS to the desired concentration, as previously described [19].

### Mouse model of melioidosis

#### Animals

Specific pathogen-free C57BL/6 mice were obtained from the Jackson Laboratory (Bar Harbor, ME). *Tlr5* ^−/−^ mice are previously described [20]. *Nlrc4* ^−/−^ mice were obtained from Vishva Dixit [21]. *Casp1* ^−/−^ *Casp11* ^−/−^ mice were obtained from Richard Flavell [22]. All mice were backcrossed at least six generations onto a C57BL/6 background and *Tlr5* ^−/−^ *Nlrc4* ^−/−^ mice were subsequently derived. Mice were housed in isolator cages with ad lib access to chow and water, and were monitored one to two times daily.

#### Infection

Mice were exposed to aerosolized bacteria in a 24 port cylindrical nose-only exposure chamber (In-Tox Products, Moriarty, NM) [19]. Aerosols were generated by a MiniHeart Hi-Flo nebulizer (Westmed, Tucson, AZ) driven at 8 L/min with 7 L/min simultaneous dilution air for 10 minutes followed by 5 minutes washout period. Pressure and airflow were controlled by an AeroMP aerosol management platform (Biaera Technologies, Frederick, MD). Bacterial deposition in each experiment was determined from quantitative culture of lung tissue from four mice sacrificed immediately after infection. Animals were examined daily for illness or death and their clinical condition recorded. When indicated, abdominal surface temperatures were measured using a Ranger MX4P digital infrared thermometer (Raytek, Santa Cruz, CA, USA). Ill animals with temperatures <21.5°C or a combination of ruffled fur, eye crusting, hunched posture and lack of resistance to handling were deemed terminal and euthanized. Spontaneous death was not required as an endpoint.

#### Bacterial quantification

Twenty four or forty eight hours after infection mice were sacrificed. The left lung and spleen each were homogenized in 1 mL sterile PBS. One hundred microliters each of homogenate and 10-fold serial dilutions were plated in duplicate on LB agar or Ashdown's medium. Colonies were counted after 2–4 days of incubation at 37°C or up to a week incubating at room temperature.

#### Lung histology and quantitative morphometry

The right lung was fixed in 4% paraformaldehyde as previously described [19]. Lung tissue was embedded in paraffin, sectioned to expose maximum surface area, and stained with hematoxylin and eosin; sections were examined by a veterinary pathologist who was blinded to group assignment. The number of focal inflammatory lesions, area of each lesion, and total lung tissue area in one representative section from each mouse were determined using Nikon NIS-Elements software.

#### Cytokine measurements

Left lung homogenates in PBS were diluted 1∶1 in lysis buffer containing 2× protease inhibitor cocktail (Roche Diagnostics, Mannheim, Germany), incubated on ice for 30 min, and then centrifuged at 1500× g. Supernatants were collected and stored at −80°C until assayed for cytokines. Whole blood was centrifuged, serum removed and stored at −80°C until assayed. IFN-γ, IL-10, IL-12p70, IL-1β, IL-6, KC, and TNF-α were measured in lung homogenates and serum using a multiplex bead assay (Luminex, Austin, TX) and reagents purchased from R&D Systems.

### Human subjects

#### Clinical cohort

Human genetic analyses were performed on patients with culture-proven melioidosis admitted to Sappasithiprasong Hospital, Ubon Ratchathani, Thailand from 1999 through 2005 [8]–[10]. A study team screening patients with clinical signs of infection cultured sputum, blood, urine and other relevant samples (for example, abscess aspirates) for *B. pseudomallei*. Samples from some patients were independently submitted for culture by hospital clinicians. Pulmonary involvement was defined by a positive *B. pseudomallei* culture from the respiratory tract or radiographic evidence of a pulmonary infiltrate and a positive *B. pseudomallei* culture from another clinical specimen. Deaths were defined as those individuals who died during their hospitalization or were discharged home in extremis for palliative care. DNA was extracted from blood using a Nucleon BACC3 kit (GE Healthcare, Buckinghamshire, UK).

### Polymorphism selection and genotyping

*NLRC4* SNP identification and selection was performed using the Genome Variation Server (http://gvs.gs.washington.edu/GVS/). Coding SNPs in the gene and haplotype-tagging SNPs were selected. Within the region encompassed by 50,000 bases upstream and downstream of *NLRC4*, SNPs with a minor allele frequency ≥2% in populations identified as Japanese, Chinese and Asian were binned into groups with R^2^≥0.8 to identify haplotype-tagging SNPs. Genotyping was performed using an allele-specific primer extension method (Sequenom Inc., San Diego, CA, USA) with reads by a MALDI- TOF mass spectrometer [8].

### Statistics

Comparisons of two and three groups of data expected to follow a normal distribution were made using Student's t test and ANOVA with a Bonferroni post-test, respectively. CFUs were log_10_ transformed before analysis. Survival analyses were performed with the log rank test. SNPs were tested for deviation from Hardy-Weinberg equilibrium using the exact test. The association of genotype with death was performed using a Chi square test or, for contingency tables with cell counts <10, the exact test. For multivariate analysis of genetic associations, logistic regression was performed adjusting for age, gender, diabetes, renal disease, or liver disease. A conservative Bonferroni correction was not performed as the variants are unlikely to be independent. Effect modification was assessed by testing the incorporation of an interaction variable into the regression model, using the likelihood ratio test. Statistics were performed with GraphPad Prism 5.0f (San Diego, CA) or Stata 11.2 (College Station, TX). A two sided p value of ≤0.05 was considered significant.

## Results

**Figure 1:**
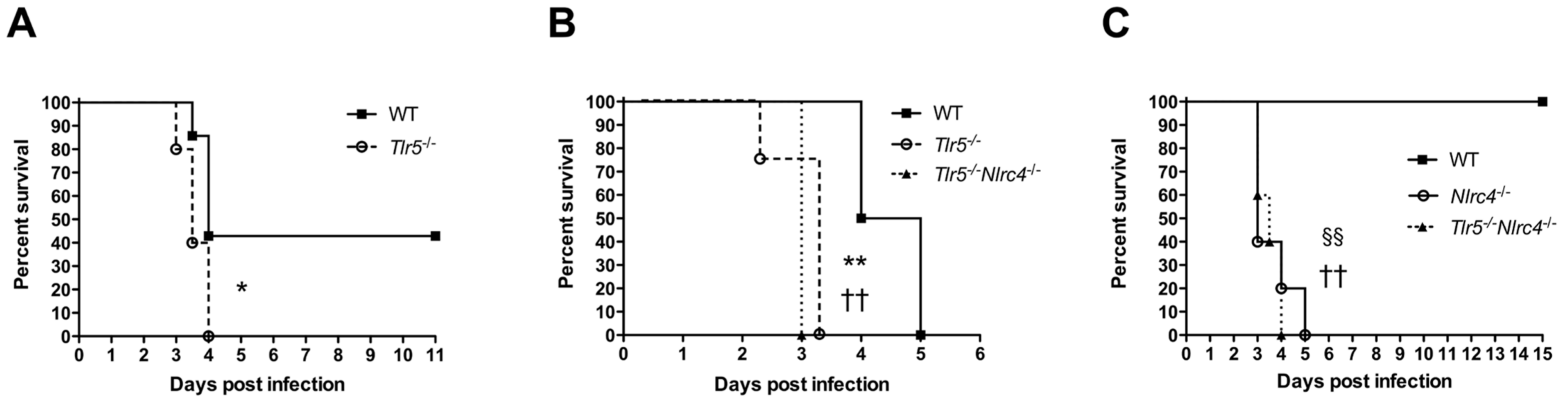
*Tlr5* and *Nlrc4* independently contribute to survival in respiratory melioidosis. Wild type (WT), *Tlr5* ^−/−^, *Tlr5* ^−/−^ *Nlrc4* ^−/−^, or *Nlrc4* ^−/−^ mice were infected with A) 361 CFU/lung B) 400 CFU/lung or C) 91 CFU/lung aerosolized *B. pseudomallei* 1026b and monitored at least daily for survival. N = 5–7 per group (A), 4 per group (B), and 5 per group (C). *, p≤0.05 and **, p≤0.01 for *Tlr5* ^−/−^ compared to wild type mice;). ††, p≤0.01 for *Tlr5* ^−/−^ *Nlrc4* ^−/−^ compared to wild type mice; §§, p≤0.01 for *Nlrc4* ^−/−^ compared to wild type mice. No significant differences in survival were noted for *Tlr5* ^−/−^ or *Nlrc4* ^−/−^ compared to *Tlr5* ^−/−^ *Nlrc4* ^−/−^ mice.

Given our previous identification of a strong association between a nonsense *TLR5* polymorphism that renders TLR5 insensitive to flagellin and survival from melioidosis [9], we examined whether the presence of *Tlr5* in murine melioidosis altered survival. We infected mice with 361 CFU *B. pseudomallei* per lung, a dose that approximates the median lethal dose (Figure 1A). We found that *Tlr5* ^−/−^ mice had significantly poorer survival than wild type mice, a phenotype that contrasts with that observed in *Tlr2* ^−/−^ or *Tlr4* ^−/−^ mice [23].

We next asked how the absence of *Nlrc4* modulated this phenotype. We infected *Tlr5* ^−/−^ and *Tlr5* ^−/−^ *Nlrc4* ^−/−^ mice with a similar dose (400 CFU/lung) of *B. pseudomallei* but found no difference in survival between mouse strains (Figure 1B). This finding suggested that the absence of flagellin sensing at the cell surface sufficiently impaired the host response such that impaired cytosolic detection of the pathogen did not substantially impact survival further. We then tested whether lack of *Nlrc4* alone altered survival in respiratory melioidosis, and how this differed from combined deficiency of *Tlr5* and *Nlrc4*. To increase the sensitivity of our model, we chose a lower inoculum that is non-lethal to wild type mice (91 CFU/lung). We found that *Nlrc4*-deficient mice were more susceptible to melioidosis than wild type mice, consistent with results from Ceballos-Olvera [24], but that there was no difference in survival between *Nlrc4* ^−/−^ mice and *Tlr5* ^−/−^ *Nlrc4* ^−/−^ mice (Figure 1C). Together, these experiments demonstrate that TLR5 and NLRC4 each contribute to host defense in murine respiratory melioidosis.

**Figure 2:**
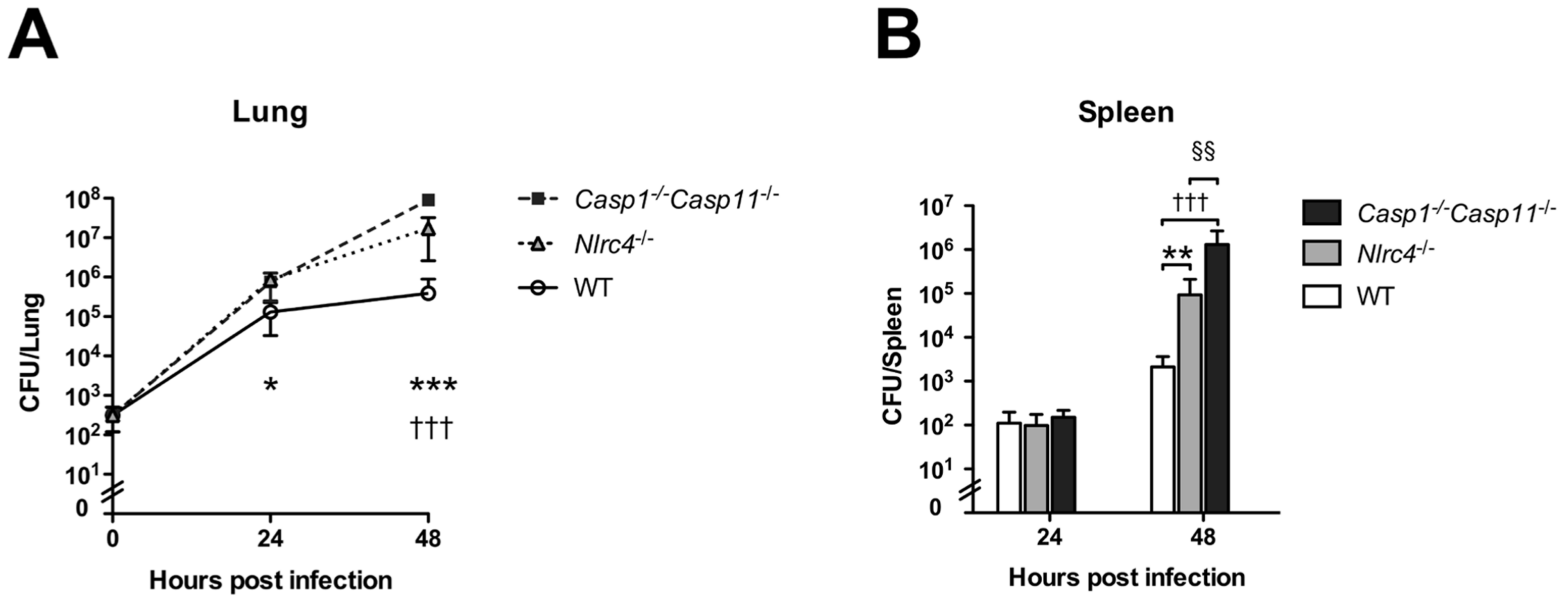
*Nlrc4* contributes substantially to *Caspase-1/Caspase-11*-dependent restriction of *B. pseudomallei* replication in the lung. Wild type (WT), *Nlrc4* ^−/−^, and *Casp1* ^−/−^ *Casp11* ^−/−^ mice were infected with 314 CFU/lung aerosolized *B. pseudomallei* 1026b. Lungs (A) and spleens (B) were harvested and quantitatively cultured 24 and 48 hours after infection. Data displayed are means ± SD and represent one of two comparable experiments. N = 4 per group per timepoint except N = 3 for *Casp1* ^−/−^ *Casp11* ^−/−^ mice at 24 h. *, p≤0.05, **, p≤0.01, and ***, p≤0.001 for *Nlrc4* ^−/−^ compared to wild type mice; †††, p≤0.001 for *Casp1* ^−/−^ *Casp11* ^−/−^ compared to wild type mice; §§§, p≤0.001 for *Nlrc4* ^−/−^ compared to *Casp1* ^−/−^ *Casp11* ^−/−^ mice.

Caspase-11 has recently been identified as a component of the noncanonical, caspase-1-independent inflammasome. We and others have found that *Casp1* ^−/−^ *Casp11* ^−/−^ mice infected with *B. pseudomallei* by the respiratory route failed to control infection (unpublished data, [24], [25]). To examine the effects of NLRC4 relative to other caspase-1- and caspase-11- dependent inflammasomes, we directly compared bacterial burdens in organs of wild type, *Nlrc4* ^−/−^, or *Casp1* ^−/−^ *Casp11* ^−/−^ mice infected with *B. pseudomallei*. Twenty four hours after an inoculum of 314 CFU/lung, bacterial growth in the lungs of both *Nlrc4* ^−/−^ and *Casp1* ^−/−^ *Casp11* ^−/−^ mice was about 0.87 log_10_ CFU greater than in wild type mice, and there was no significant difference between CFU in *Nlrc4* ^−/−^ compared to *Casp1* ^−/−^ *Casp11* ^−/−^ mice (Figure 2). Forty eight hours after infection, bacterial growth in the lungs of both *Nlrc4* ^−/−^ and *Casp1* ^−/−^ *Casp11* ^−/−^ mice had increased significantly compared to wild type mice (by 1.87 log_10_ CFU and 2.69 log_10_ CFU, respectively). Despite a trend towards greater pulmonary bacterial burdens in *Casp1* ^−/−^ *Casp11* ^−/−^ mice than in *Nlrc4* ^−/−^ mice, this did not reach statistical significance. Bacterial burdens in the spleens are an indication of dissemination beyond the pulmonary compartment. Although bacteria were detectable 24 hours after infection, there were no significant differences between the three mouse strains. Forty eight hours after infection, CFU were significantly greater in *Casp1* ^−/−^ *Casp11* ^−/−^ mice compared to *Nlrc4* ^−/−^ mice which in turn had greater bacterial burdens compared to wild type mice. These data confirm that while deficiency of both caspase-1 and caspase-11 severely impairs control of *B. pseudomallei* replication in the lung, NLRC4 accounts for much of the inflammasome-dependent phenotype [24].

**Figure 3:**
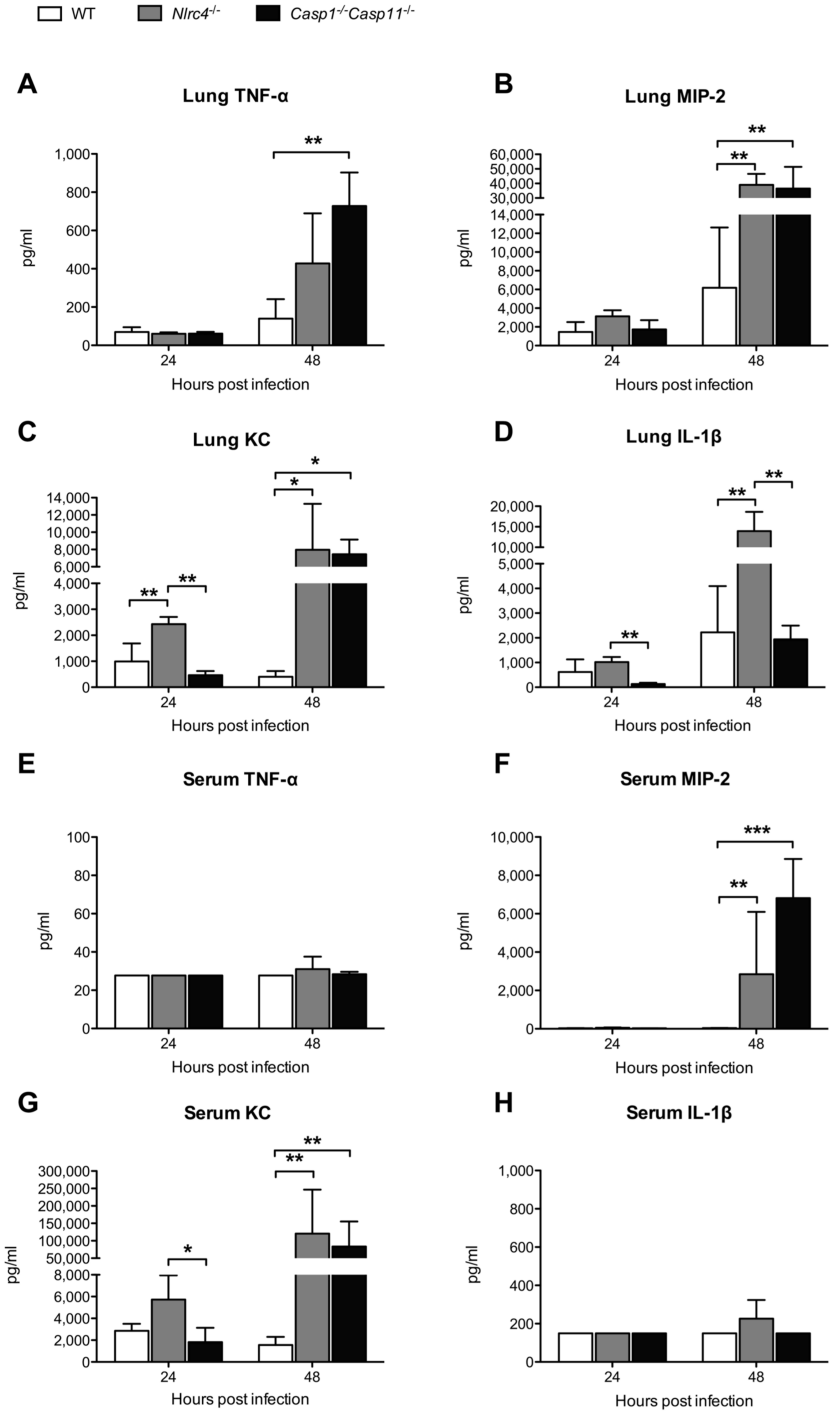
Differential *Nlrc4- and Caspase/Caspase11*-dependent lung and serum cytokine responses in respiratory melioidosis. Wild type (WT), *Nlrc4* ^−/−^, and *Casp1* ^−/−^ *Casp11* ^−/−^ mice were infected with 314 CFU/lung aerosolized *B. pseudomallei* 1026b. TNF-α, MIP-2, KC, and IL-1β were measured in lung homogenate (A–D) and serum (E–H) 24 and 48 hours after infection. Data displayed are means ± SD and represent one of two comparable experiments. N = 4 per group per timepoint except N = 3 for *Casp1* ^−/−^ *Casp11* ^−/−^ mice at 24 h. *, p≤0.05. **, p≤0.01, ***, p≤0.001.

We next evaluated selected cytokine and chemokine responses in the lungs of these mice (Figure 3). There were no differences in TNF-α or MIP-2 levels between mouse strains at 24 hours. As expected, IL-1β was very low in *Casp1* ^−/−^ *Casp11* ^−/−^ mice but was not impaired in *Nlrc4* ^−/−^ mice. Chemokine KC was higher in *Nlrc4* ^−/−^ mice compared to wild type and to *Casp1* ^−/−^ *Casp11* ^−/−^ mice. By 48 hours after infection, TNF-α levels in *Casp1* ^−/−^ *Casp11* ^−/−^ mice were significantly greater than wild type. MIP-2 and KC levels in *Casp1* ^−/−^ *Casp11* ^−/−^ and *Nlrc4* ^−/−^ mice were higher than in wild type mice. IL-1β was elevated in all mice compared to 24 hour levels, but was significantly elevated in *Nlrc4* ^−/−^ mice in comparison to wild type and to *Casp1* ^−/−^ *Casp11* ^−/−^ mice. In serum 24 hours after infection, TNF-α, MIP-2, and Il-1β levels were low but KC was readily detectable and higher in *Nlrc4* ^−/−^ mice compared to *Casp1* ^−/−^ *Casp11* ^−/−^ mice. At 48 hours, despite higher bacterial burdens in the spleens of *Nlrc4* ^−/−^ and *Casp1* ^−/−^ *Casp11* ^−/−^ mice compared to wild type mice, serum TNF-α and IL-1β remained uniformly low. In contrast, MIP-2 and KC levels increased substantially in both *Nlrc4* ^−/−^ and *Casp1* ^−/−^ *Casp11* ^−/−^ mice. In line with previously published data [24], these results point to non-NLRC4-mediated pathways of IL-1β production in the lung, but suggest that systemically, NLRC4 mediates TNF-α and IL-1β but not MIP-2 or KC release.

**Figure 4:**
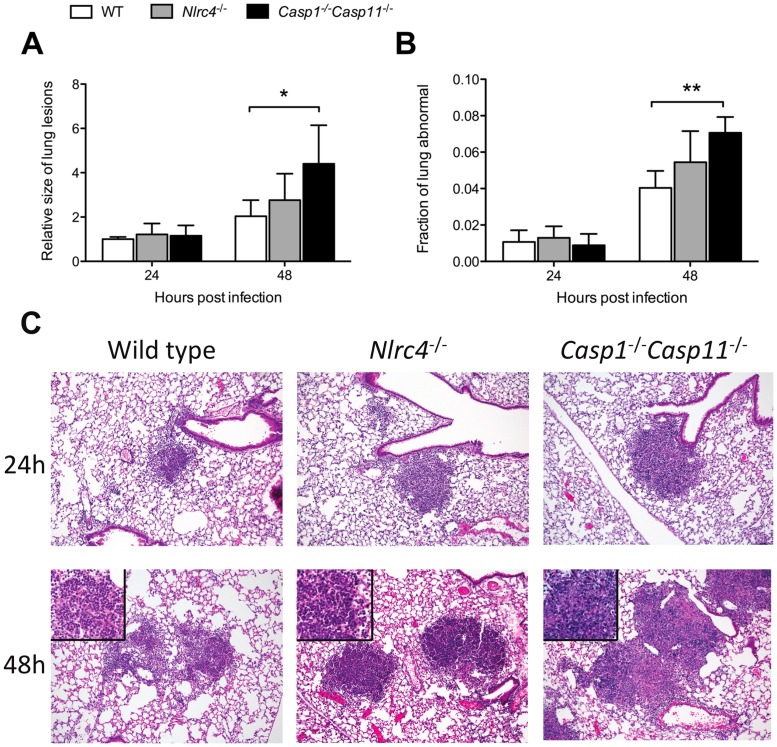
*Nlrc4- and Caspase-1/Caspase-11*-dependent histologic changes in the lung during respiratory melioidosis. Wild type (WT), *Nlrc4* ^−/−^, and *Casp1* ^−/−^ *Casp11* ^−/−^ mice were infected with 314 CFU/lung or 194 CFU/lung aerosolized *B. pseudomallei* 1026b in two separate experiments. Lungs were fixed and stained with hematoxylin and eosin 24 and 48 hours after infection. The average size of each focal pulmonary infiltrate normalized to measures from wild type mice at 24 hours (A) and the fraction of total lung area that was abnormal (B) were quantified for each group of mice. Data displayed are means ± SD from the two experiments. N = 4–7 per group per timepoint. *, p≤0.05, **, p≤0.01. (C) Representative histologic features of pulmonary infection with *B. pseudomallei*. At 24 h post-infection lesions are similar between the groups in pattern, distribution and size but have slightly varying morphologic features. Inflammatory foci in WT mice are composed of moderately dense aggregates of nearly pure, intact neutrophils that fill vessels and alveolar spaces but preserve architectural elements. In contrast focal inflammatory cell aggregates in *Nlrc4* ^−/−^ and *Casp1* ^−/−^ *Casp11* ^−/−^ while also predominately neutrophilic (with few mononuclear cells) are much more densely packed, slightly obscuring some normal structures and, in the case of *Casp1* ^−/−^ *Casp11* ^−/−^ showing early evidence of nuclear fragmentation. By 48 hours post-infection phenotypic differences are more distinct. Focal lesions within WT mice while modestly larger remain predominately neutrophilic with limited evidence of parenchymal tissue necrosis and relatively mild neutrophil condensation and fragmentation. Nuclear condensation and fragmentation is a hallmark of lesions in *Nlrc4* ^−/−^ mice at 48 h but parenchymal injury remains relatively modest with many alveolar wall outlines preserved and small airway involvement very limited. Mixed inflammatory cells surround these lesions. This contrasts with the severe injury evident in *Casp1* ^−/−^ *Casp11* ^−/−^ mice at 48 h. In these mice lesions are substantially larger and efface all or most parenchymal and vascular structures which are focally replaced by necrotic cells and a dark pink coagulum containing variably fine nuclear debris. This debris commonly extends into small airways. Intact inflammatory cells of any type are rarely evident within these lesions although adjacent vessels are surrounded by small numbers of intact mononuclear cells.

Inhalation of *B. pseudomallei* results in scattered, dense cellular pulmonary infiltrates [19]. Histopathologic examination of the lungs of *Nlrc4* ^−/−^ and *Casp1* ^−/−^ *Casp11* ^−/−^ mice 24 hours after airborne infection with *B. pseudomallei* showed relatively similar sized neutrophilic infiltrates and percent of lung involved in these mice compared to wild type mice although there was minor variation in morphologic features, such as earlier evidence of nuclear fragmentation in *Casp1* ^−/−^ *Casp11* ^−/−^ mice (Figure 4). However, at 48 hours, inflammation was more severe, particularly in *Casp1* ^−/−^ *Casp11* ^−/−^ mice, which displayed larger and necrotic parenchymal lesions that lacked identifiable intact inflammatory cells.

We have found that a human genetic polymorphism in *TLR5* is associated with outcome from melioidosis [9]. Given the clear role for *Nlrc4* in murine respiratory melioidosis, we investigated whether human genetic variation in the *NLRC4* region was associated with death in human respiratory melioidosis. We genotyped five *NLRC4* region single nucleotide polymorphisms (SNPs) (rs455060, rs212703, rs410469, rs462878, and rs6757121) selected as described in the methods in 173 melioidosis patients with clinical evidence of pulmonary involvement. The call rate for four SNPs was above 97.5%; one (rs212703) was discarded due to a low call rate. Fifty eight of the 173 subjects (34%) died. In survivors, no variant deviated from Hardy-Weinberg equilibrium. rs6757121 was associated with protection against death in a general genetic model, p = 0.012 (Table 1). Adjusting for age, sex, and pre-existing conditions, the effect was strongest in a dominant model [odds ratio (OR) 0.35, 95% CI:0.13–0.91, p = 0.03]. rs6757121 is located about 0.3 kb downstream of *NLRC4* and occurs with a minor allele frequency of 10%.

**Table 1:** Association of *NLRC4* region variants with death in respiratory melioidosis.

Variant	Genotype	Death		P
		Yes	No	
rs455060				
	CC	24	55	
	CT	24	46	0.50
	TT	10	13	
		HWE P = 0.52		
rs410469				
	TT	13	28	
	TG	30	47	0.46
	GG	15	37	
		HWE P = 0.13		
rs462878				
	AA	17	33	
	AG	29	53	0.88
	GG	12	27	
		HWE P = 0.57		
rs6757121				
	CC	50	85	
	CT	5	28	0.012
	TT	1	0	
		HWE P = 0.21		

Hardy-Weinberg P values calculated by the exact test.

General genetic model P values calculated by the Chi square test or, for cell counts <10, Fisher's exact test.

We next tested whether our previously reported association between *TLR5*_1174C>T_ – a nonsense polymorphism that truncates the receptor in the extracellular domain rendering it non-responsive to flagellin – and survival in melioidosis [9] is also seen in the subset of melioidosis patients with respiratory disease. We found that the adjusted OR of death was 0.14, 95% CI 0.03–0.64, p = 0.01. To determine whether co-inheritance of this *TLR5* variant and the *NLRC4* region variant rs6757121 alters the risk of death from respiratory melioidosis, we assessed the effect of including both together in the model. The OR of death for each variant remained unchanged, although the effect of a cross-product interaction term could not be determined due to 100% survival in carriers of both variants. The estimated OR of death for carriers of both variants was 0.04, 95% CI: 0.006–0.27, p = 0.001 (Table 2). Together, these data show that the *NLRC4* and *TLR5* variants are each associated with survival and that co-inheritance of the variants has an additive but not synergistic effect.

**Table 2:** Association of co-inheritance of *NLRC4* rs6757121 and *TLR5*_1174C>T_ with death in respiratory melioidosis.

rs6757121	*TLR5*_1174C>T_	Death		OR (95%CI)	P
		Yes	No		
0	0	48 (42%)	66 (58%)	reference	
1	0	6 (21%)	23 (79%)	0.32 (0.12–0.86)	0.02
0	1	2 (10%)	18 (90%)	0.13 (0.03–0.61)	0.009
1	1	0 (0%)	5 (100%)	0.04 (0.006–0.27)	0.001

1 and 0 indicate the presence or absence of the dominant genotype for each variant.

P values calculated by logistic regression.

## Discussion

The results of our investigations show that NLRC4 and TLR5, key components of two flagellin sensing pathways, each contributes to host defense in murine respiratory melioidosis. We did not detect any additional impact of deficiency of both *Nlrc4* and *Tlr5* on survival. Furthermore, NLRC4 is responsible for much of the failure of pulmonary bacterial containment seen in caspase-1/-11-deficient mice. In humans, we show that an *NLRC4* genetic variant is associated with survival in respiratory melioidosis, and there is an additive effect of co-inheritance of risk variants in *TLR5* and *NLRC4*.

Recent investigations have demonstrated that NLRC4 is involved in recognition of several bacterial ligands such as components of the T3SS or flagellin, and this specificity is determined by various NAIPs [13]–[16]. In contrast, the only reported ligand of TLR5 is flagellin [4]. *B. pseudomallei* activates TLR5 [9] and aflagellated *B. pseudomallei* induces impaired TLR5-dependent NF-κB activation in vitro [unpublished data]. Our present results show that *Tlr5* ^−/−^ mice are more susceptible to *B. pseudomallei* in a model of respiratory infection, in contrast to deficiency in *Tlr2*, which actually confers resistance, or *Tlr4*, which has no apparent effect on survival [23]. Although MyD88 is an adapter molecule for all three of these TLRs, mice deficient in *Myd88* show a similar phenotype to deficiency in *Tlr5* after respiratory infection with *B. pseudomallei* [26]. Flagellin-sensing appears to be a crucial element of host defense in murine respiratory melioidosis. However, we have not observed significant impairment in TNF-α production from *Tlr5* ^−/−^ alveolar macrophages stimulated ex vivo with killed *B. pseudomallei* [unpublished data] and it is notable that our studies of murine respiratory infection with *B. thailandensis* (a related and flagellated but less virulent organism) have not shown any *Tlr5*-dependent phenotype [20]. Thus, in vitro data, and infections with model organisms may not fully recapitulate the complexity of in vivo infections with fully virulent *B. pseudomallei*.

Like TLR5, NLRC4 appears to play a central role in host defense in respiratory murine melioidosis. Interestingly, while NLRC4 detects flagellin from many bacterial species, it appears to not detect *B. thailandensis* (and presumably *B. pseudomallei*) flagellin [14], thus, the effect of NLRC4 in vivo may be attributable to T3SS sensing. *B. pseudomallei* expresses several T3SSs [27] and T3SS3 facilitates virulence in a number of ways [28]–[31]. The *B. pseudomallei* T3SS rod and needle proteins BsaK and BsaL, respectively, are detected in an NLRC4-dependent fashion in mice [15], [32]. Recent work by Bast et al demonstrates the importance of BsaK for NLRC4-dependent caspase-1 activation in *B. pseudomallei*-infected macrophages and for virulence in murine melioidosis [33]. Intriguingly, despite the different sensing functions of TLR5 and NLRC4, absence of only one sensor imparts significant clinical impairment; there is no additive effect on survival of combined *Tlr5* and *Nlrc4* deficiency in murine melioidosis, even at doses that are non-lethal to wild type mice.

NLRC4 is just one of many pathogen recognition receptors that activate the caspase-1-dependent inflammasome. The inflammasome processes pro-IL-1β and pro-IL-18 to their active forms and also induces pyroptosis, a caspase-1-dependent lytic cell death pathway. In our studies, *Nlrc4* ^−/−^ mice did not show a significant difference compared to *Casp1* ^−/−^ *Casp11* ^−/−^ mice with respect to bacterial replication in the lung following respiratory infection, but did show a difference in disseminated infection to the spleen, consistent with the work of Ceballos-Olvera et al [24]. This difference in dissemination may be due to caspase-11, which also has been implicated in defense against *B. pseudomallei* [18]. Relative to *Casp1* ^−/−^ *Casp11* ^−/−^ mice, *Nlrc4* ^−/−^ mice showed preserved pulmonary IL-1β production. These data raise the possibility that much of the early effect of inflammasome-dependent control of bacterial replication in the lung is primarily NLRC4-dependent and the function of NLRC4 may be due to pyroptosis or as-yet-undefined roles of NLRC4 rather than cytokine processing. It may be that a secondary canonical inflammasome, perhaps NLRP3, responds to *B. pseudomallei* infection only once bacterial burdens become extremely high, resulting in the observed IL-1β secretion. These observations are concordant with the work by Ceballos-Olvera et al, who additionally showed that processing of pro-IL1β to the active form was not impaired in *Nlrc4* ^−/−^ bone marrow-derived macrophages or in the bronchoalveolar lavage fluid of *Nlrc4* ^−/−^ mice infected with *B. pseudomallei* [24]. Furthermore, despite differences in experimental methods and timing, the histology of lungs from *Nlrc4* ^−/−^ mice infected with *B. pseudomallei* in our study appeared comparable to that of wild type mice treated with IL-1β and infected with *B. pseudomallei* by Ceballos-Olvera et al [24]. Notably, however, we found that systemic IL-1β and TNF-α levels were almost undetectable in *Nlrc4* ^−/−^ mice, despite high bacterial burdens in the spleen. This contrasted with high MIP-2 and KC concentrations in the serum, suggesting that there may be distinctly different regulatory effects of NLRC4 in various compartments.

Our data also demonstrate that NLCR4 inflammasome-dependent innate immune signaling is not the same for *B. pseudomallei* as other Gram-negative pulmonary pathogens. Respiratory infection with *Legionella pneumophila*, another Gram-negative, flagellated, intracellular pathogen is also restricted by NLRC4 and this effect is dependent on the presence of flagellin [21], [34]. However, following *L. pneumophila* infection there was no difference in bronchoalveolar lavage fluid cell counts or in lung cytokine levels of *Nlrc4^−/−^* mice compared to wild type mice, although there was greater histologic inflammation in the lungs of *Nlrc4^−/−^* mice [21]. As in *B. pseudomallei* infection, *Nlrc4* ^−/−^ mice are more susceptible to *Klebsiella pneumoniae* (a non-flagellated, extracellular pathogen) infection by the pulmonary route, with greater bacterial replication in the lungs, dissemination to the spleen, and death [35] although this effect was not observed at higher doses [36]. In contrast to our findings, pulmonary inflammation as assessed by TNF-α, KC, IL-1β, and MIP-2 levels and histologic score is reduced in *Nlrc4* ^−/−^ mice infected with *K. pneumoniae* [35]. These differences may be due to the presence of flagellin or the intracellular nature of *B. pseudomallei*, or to the apparent lack of NLRC4-mediated pyroptosis induced by *K. pneumoniae* [35].

Our human genetic study provides adjunctive evidence for the importance of NLRC4 in respiratory melioidosis although it requires validation. Few clinically associated polymorphisms in *NLRC4* have been described thus far and the function of rs6757121 is otherwise unknown. We have previously reported the association of variation in *TLR5* with survival in melioidosis regardless of site of infection and here show that the association holds in respiratory disease [9], [10]. Unlike in mice, modeling suggests that co-inheritance of variants in *NLRC4* and in *TLR5* increases the effect in an additive manner. Another important difference between mice and humans is that in humans, blunting of TLR5 function – as found in carriers of a nonsense polymorphism – is in fact protective against death from melioidosis [9], [10]. This seemingly opposite phenotype from mice underscores the challenges of mimicking human sepsis in mice [37]–[39].

In conclusion, we show that NLRC4 and TLR5 are essential elements of host defense in murine respiratory melioidosis, and that genetic variation in these genes is associated with outcome from human respiratory melioidosis.
